# A Systematic Review of Modeling and Simulation for Precision Diamond Wire Sawing of Monocrystalline Silicon

**DOI:** 10.3390/mi15081041

**Published:** 2024-08-17

**Authors:** Ansheng Li, Hongyan Wang, Shunchang Hu, Yu Zhou, Jinguang Du, Lianqing Ji, Wuyi Ming

**Affiliations:** 1 Mechanical and Electrical Transportation Science and Education Center, Huanghe Science and Technology University, Zhengzhou 450006, China; liansheng@zzuli.edu.cn; 2 Mechanical and Electrical Engineering Institute, Zhengzhou University of Light Industry, Zhengzhou 450002, China; hongyanwang923@163.com (H.W.); hushunchang2022@gmail.com (S.H.); dujinguangzzuli@163.com (J.D.); 3 Guangdong Provincial Key Laboratory of Digital Manufacturing Equipment, Huazhong University of Science and Technology, Dongguan 523808, China; 4 Gokin Solar Company Limited, Zhuhai 519031, China; yu.zhou@gokinsolar.com

**Keywords:** mathematical analytical model, molecular dynamics, finite element methods, diamond wire sawing, monocrystalline silicon

## Abstract

Precision processing of monocrystalline silicon presents significant challenges due to its unique crystal structure and chemical properties. Effective modeling and simulation are essential for advancing the understanding of the manufacturing process, optimizing design, and refining production parameters to enhance product quality and performance. This review provides a comprehensive analysis of the modeling and simulation techniques applied in the precision machining of monocrystalline silicon using diamond wire sawing. Firstly, the principles of mathematical analytical model, molecular dynamics, and finite element methods as they relate to monocrystalline silicon processing are outlined. Subsequently, the review explores how mathematical analytical models address force-related issues in this context. Molecular dynamics simulations provide valuable insights into atomic-scale processes, including subsurface damage and stress distribution. The finite element method is utilized to investigate temperature variations and abrasive wear during wire cutting. Furthermore, similarities, differences, and complementarities among these three modeling approaches are examined. Finally, future directions for applying these models to precision machining of monocrystalline silicon are discussed.

## 1. Introduction

Due to the swift progression of socioeconomic factors and technology, the utilization of rigid and fragile materials like silicon, silicon carbide, and monocrystalline silicon has grown in a variety of domains, encompassing aviation, aerospace, military applications, integrated circuits, and photovoltaic (PV) [1,2,3]. Silicon, one of the most abundant semiconductor elements in the Earth’s crust, offers the advantages of easy purification, extended longevity, stable performance, and high reliability compared to other semiconductor materials [4]. It stands as one of the most accessible materials with the highest purity, rendering it exceptionally suitable for the development, production, and application of solar cells [5,6]. Silicon-based solar cells predominantly include amorphous silicon, monocrystalline silicon, and polycrystalline silicon cells, among other types [7]. In the realm of PV, crystalline silicon materials have emerged as the primary choice for solar cell production and application owing to their ample reserves, ongoing enhancements in the conversion efficiency of crystalline solar cells, and diminishing manufacturing costs [8,9]. Monocrystalline silicon has found extensive use in the integrated circuit and PV sectors, thanks to its exceptional semiconductor properties [8]. Globally, monocrystalline silicon wafers account for more than 90% of the substrates employed in integrated circuits [10,11].

### 1.1. Applications of Monocrystalline Silicon in PV Modules

**Figure 1:**
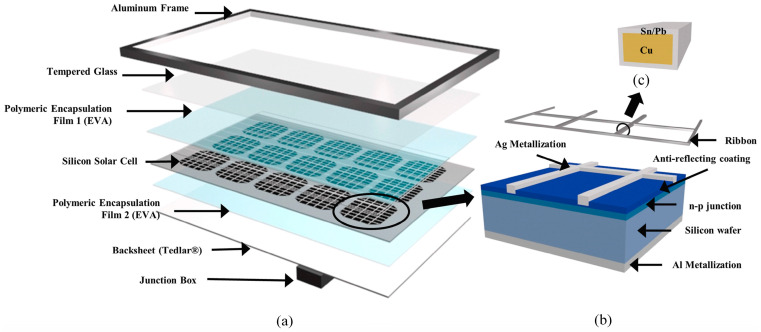
Structural composition of PV panels [12]: (**a**) schematic diagram of the solar PV panel structure, (**b**) PV cells composition diagram, (**c**) solder ribbon construction diagram.

Monocrystalline silicon PV modules are widely recognized for their attributes, including robustness, durability, and high efficiency. Figure 1 illustrates the structure of these modules. In Figure 1a, the configuration of a monocrystalline silicon PV module is akin to that of a sandwich, featuring an outer protective layer made of an Al alloy frame [12]. At the base, a junction box is responsible for converting, storing, and transmitting the harvested energy. The layers within this sandwich, ordered from top to bottom, consist of reinforced glass, a polymeric encapsulant, silicon-based solar cells, another polymeric encapsulant, and the backsheet. Normally, an encapsulation material like ethylene vinyl acetate is employed to bond the solar cells to the tempered glass and backsheet. This offers safeguarding against heat, mechanical stress, ultraviolet rays, and moisture [13]. The backsheet is commonly composed of laminated sheets made from various polymer materials [14]. Numerous backsheet materials primarily or partly comprise fluorinated polymers, including polyvinyl fluoride, polyvinylidene fluoride, or ethylene chlorotrifluoroethylene [15]. The primary component responsible for power generation in monocrystalline silicon PV modules is the solar cell, typically with a thickness of about 200 μm, as seen in Figure 1b. To create an n-p junction for generating photovoltage, either boron or phosphorus is used to dope the silicon wafer. Additionally, an anti-reflective layer is applied to the upper surface of the wafer to minimize light reflection from the silicon, thus enhancing the efficiency of light absorption and conversion within the PV panel. The primary components of this anti-reflective layer include SiO, SiO_2_, Si_3_N_4_, and Al_2_O_3_ [16]. Silver wire electrodes adorn the top layer of the solar cell, while the bottom electrode is completely coated with aluminum [17]. For interconnecting the monocrystalline silicon cells, solder ribbons are commonly used, primarily comprising copper, as illustrated in Figure 1c.

### 1.2. Methodology

The traditional process for cutting monocrystalline silicon primarily relies on the interaction between the silicon’s lattice structure and the mechanical operation of the cutting tool [18,19]. In the dicing process, a cutting tool applies force to the monocrystalline silicon, causing it to fracture along the lattice plane to produce the desired wafer. The connection between the conventional processing of monocrystalline silicon and the diamond wire sawing (DWS) mechanism is rooted in their shared dependence on the physical and mechanical properties of monocrystalline silicon for cutting. DWS can be viewed as an advanced and optimized iteration of traditional cutting techniques. DWS technology has found extensive use in slicing monocrystalline silicon for PV applications. In response to the precision slicing requirements of silicon wafers in semiconductor manufacturing, significant advancements have been achieved in wire cutting technology. This method enables the production of thin wafers with micron-level warping, uniform thickness, and minimal edge loss [20,21]. By introducing sustainable and efficient technologies into various material processing techniques, the surface quality of wafers can be improved. This can promote technological advancements across different fields, leading to more efficient and environmentally friendly manufacturing processes [22,23]. By understanding the processing mechanisms involved in removing brittle materials from monocrystalline silicon and generating surfaces, it becomes possible to establish a mathematical model for DWS. Subsequent numerical calculations of the sawing process enable the analysis and validation of the effectiveness of experimental models [24].

**Figure 2:**
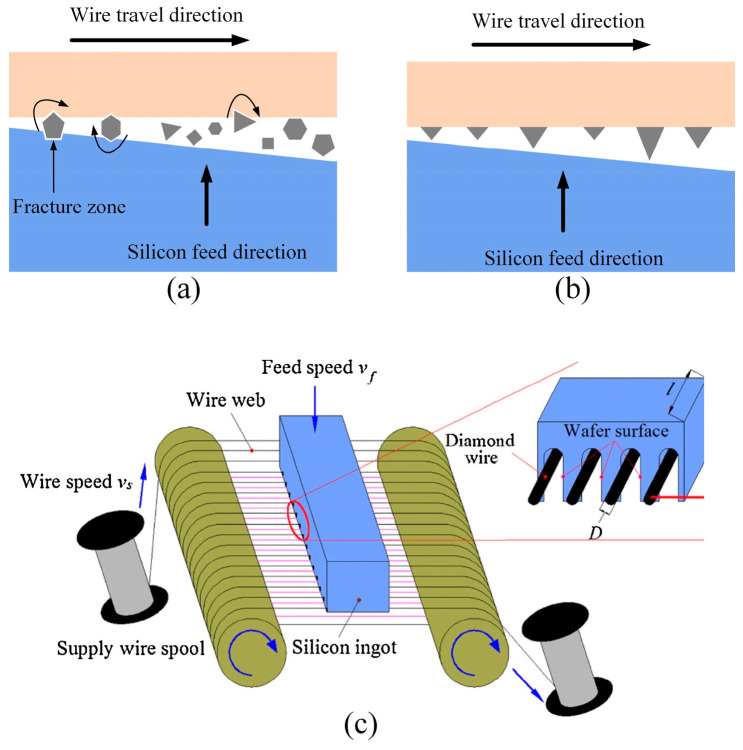
Schematic diagram of the material removal pattern: (**a**) free abrasive cut; (**b**) fixed abrasive cut; (**c**) schematic diagram of cutting silicon ingots with fixed abrasive DWS [22].

The DWS technique employs either unidirectional or reciprocating motion of the diamond wire to establish a relative grinding motion between the wire and the target material, as depicted in Figure 2. This motion serves the purpose of cutting. In Figure 2a, the application of a diluted abrasive liquid mixture for slicing delicate silicon wafers is depicted. During this procedure, sturdy SiC particles, suspended within a solution of polyethylene glycol, facilitate the cutting process. Particles of sand incorporated within the cutting groove contribute to the wafer’s slicing. The grooved channel contains these sand particles, playing a role in the wafer’s separation through a three-step material removal process. This interaction takes place between the silicon ingot and the cutting grains in this process. Conversely, the fixed abrasive DWS process, depicted in Figure 2b, shares similarities with the previously mentioned loose abrasive slurry method. The main difference lies in the utilization of abrasive particles, specifically diamonds, securely bonded to an electroplated nickel-coated steel wire. Furthermore, a cutting fluid based on water is used instead of the polyethylene glycol slurry. In contrast to the trilateral material removal observed in the conventional mortar steel wire cutting method, DWS employs a dual-component material removal mechanism. This mechanism functions between the steel wire-bound diamond particles and the material being cut. Notably, there is no rolling motion experienced by the abrasive grains. Consequently, this enhances cutting efficiency significantly, surpassing that of the trilateral material removal mode. Figure 2c [22] provides a visual representation of the DWS process. The length of contact between the silicon ingot and the diamond wire is labeled as *l*, while the diamond wire’s diameter is indicated as *D*. It is important to highlight that the contact length l precisely corresponds to the length of the silicon ingot. During this procedure, the diamond wire moves axially at a speed designated as *v_s_*, while the silicon ingot is fed perpendicular to the diamond wire grid at a velocity of *v_f_*.

Monocrystalline silicon processing holds significant importance due to its wide-ranging applications in fields like solar cells and optoelectronic devices. Its remarkable electrical and optical properties establish it as a crucial material in contemporary technology. Processing monocrystalline silicon is a multifaceted procedure involving multiple stages and the fine-tuning of numerous parameters. Simulation and modeling enable the prediction of how different parameters and process choices affect the final product quality and output, all before actual processing occurs [25,26]. Consequently, this allows for workflow optimization and cost reduction by avoiding trial and error. Additionally, simulation and modeling provide a deeper insight into material behavior and the processing sequence, thereby aiding in the refinement of device design and process flow [27,28,29]. When compared to other techniques, such as single-point diamond turning and diamond grinding of monocrystalline silicon, DWS poses distinct challenges in modeling. These challenges arise from the collective action of numerous diamond particles embedded within a thin wire [30,31]. By providing insights into the intricate interactions involved in DWS, simulation and modeling are crucial for improving product quality, optimizing process parameters, and advancing DWS technology.

DWS is a common processing method used to cut monocrystalline silicon. The basic concept of this technique is to execute cuts in monocrystalline silicon by means of diamond particles embedded in a thin rope. The basic principles of DWS are relatively intuitive, but the process is relatively complex in practice. By reviewing the applications of the mathematical analysis model (MAM), molecular dynamics model (MD), and finite element method (FEM) model in analyzing DWS and diamond grinding of monocrystalline silicon, a more profound comprehension of the mechanisms involved in monocrystalline silicon wire cutting has been attained. DWS uses a multi-particle cutting mechanism, which presents problems and modeling complications distinct to those of diamond turning with single-point diamond tools. Furthermore, even though material removal mechanisms and tool wear are two areas where diamond grinding and DWS are similar, DWS’s distributed cutting action calls for different simulation techniques. Through these machining methods, a series of information about the cutting mechanism, cutting parameters, tool performance, and machining effect can be obtained, which are all instructive for understanding and optimizing the DWS process. This knowledge is combined with the special needs and process characteristics of wire cutting in order to more fully understand and improve DWS technology.

### 1.3. Review Structure

The subsequent sections of this review are organized as follows: Section 2 revisits the mathematical modeling process in the context of monocrystalline silicon processing. Mathematical modeling enables a comprehensive examination of the physical mechanisms at play in monocrystalline silicon processing. Section 3 reviews the process of using MD in monocrystalline silicon processing. MD simulations model atomic behavior within monocrystalline silicon, providing profound insights into the material’s microstructure and properties. This enables the revelation of atomic interactions and material transformations during processing, forming a fundamental understanding crucial for process optimization. Section 4 outlines the utilization of FEM analysis in monocrystalline silicon processing. FEM analysis can simulate the temperature distribution and abrasive wear during wire cutting of monocrystalline silicon under different loading conditions. Analyzing these results facilitates the optimization of device structural designs, ensuring the necessary stability and reliability in real-world applications. Section 5 explores the similarities, distinctions, and complementarity among mathematical modeling, MD, and FEM analysis. Section 6 offers a perspective on the modeling and simulation processes in monocrystalline silicon processing. The main objective of this study is to improve our comprehension of how the material removal and surface generation mechanisms in monocrystalline silicon processing are influenced by process parameters, using modeling and simulation techniques. This deeper understanding will drive the advancement of high-speed precision cutting methods for silicon crystals.

## 2. Mathematical Analytical Model

### 2.1. Principles of MAM

A MAM is a methodology used for investigating problems, developing models, and solving complex scenarios through mathematical analysis and deductive reasoning. These models employ mathematical principles such as equations, formulas, and functions to uncover core issues, analyze relationships, and predict outcomes [32,33]. In monocrystalline silicon processing and engineering, MAMs serve as powerful tools for theoretical exploration, experimental design, and issue resolution [27,34]. The primary challenge in DWS of silicon, compared to germanium and silicon carbide, lies in silicon’s brittle behavior and its tendency to develop subsurface damage (SSD) [35,36]. These characteristics make silicon more prone to chipping and cracking during the cutting process, requiring precise control of cutting parameters to minimize defects. They play a crucial role in optimizing processing parameters to enhance efficiency, reduce energy consumption, and improve product quality in semiconductor device production using monocrystalline silicon [16,37].

**Figure 3:**
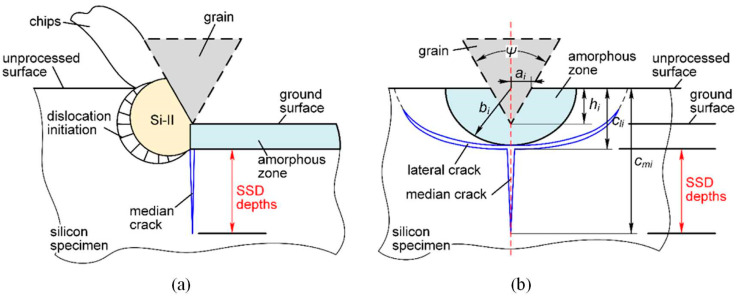
The (**a**) side and (**b**) cross section views of SSD in the silicon scratching process [27].

The distinct mechanical and physical characteristics of silicon, such as its propensity to form SSD during processing and its brittle nature, were taken into account during the MAM modeling process. In order to provide an exact representation of silicon’s properties during the cutting process, these models were created with the goal of optimizing cutting settings, minimizing surface flaws, and improving processing quality. In silicon industrial production, grinding serves as the final “rough” machining stage preceding the precision-intensive lapping phase, primarily due to its ability to achieve substantial material removal rates at a relatively low cost [38]. Grinding smooth surfaces often leads to severe SSD, including microcracks, dislocations, and stacking faults [39,40]. Research on SSD in silicon grinding originates from studies on silicon scratching, as shown in Figure 3a [27]. Subsurface behaviors during silicon scratching [41,42,43] include: (i) formation of fan-shaped amorphous zones with transverse cracks beneath the surface (Figure 3b); (ii) presence of metallic silicon (Si-II) zones and crystal dislocations due to shear stress (Figure 3a); and (iii) vertical median cracks extending downward from the amorphous zone. The flexible and loosely packed amorphous area (i) and Si-II zone (ii) are easily removed by abrasive grains during cutting, making median cracks (iii) the primary cause of SSD onset [44]. The expression for generating scratch-related SSD is provided in Equation (1) [27]:

(1) $$\mathrm{SSD}=c_{mi}-c_{li}$$

where the depths of median and lateral cracks are denoted as *c_mi_* and *c_li_*, respectively.

Gu et al. [45] established a general relationship between the median crack depth (*c_mi_*) and the grain penetration depth (*h_i_*) as follows in Equation (2):

(2) $$c_{mi}=k_{1}\cdot h_{i}^{4/3}$$

The coefficient k_1_ is defined in Equation (3) [27]:

(3) $$k_{1}=0.206\cdot {\left(\frac{\sqrt{E_{s}\cdot H_{s}}}{K_{c}\cdot \beta }\right)}^{2/3}\cdot {\left(\cot\frac{\psi }{2}\right)}^{4/9}\cdot {\left(\tan\frac{\psi }{2}\right)}^{4/3}$$

In Equation (3), *H_s_*, *β* ≈ 0.3, and K*_c_* represent the scratch hardness, elastic recovery parameter, and fracture toughness of silicon, respectively.

Since lateral cracks form at the bottom of the amorphous region, *c_li_* may approximately equal the radius *b_i_* of the fan-shaped amorphous zone. Jing et al. [46] derived the relationship, and the contact area size, between the grain and the specimen as shown in Equation (4).

(4) $$b_{i}=a_{i}\cdot \sqrt{\frac{3\left(1-2v\right)}{5-4v}+\frac{2\sqrt{3}}{\pi \left(5-4v\right)}\cdot \frac{E_{s}}{\sigma_{y}}\cdot \cot\left(\frac{\psi }{2}\right)}$$

In Equation (4), *v*, *E_s_*, and $\sigma_{y}$ denote the Poisson’s ratio, Young’s modulus, and yield stress of silicon, respectively. ψ represents the inclusion angle of the grain, and a_i_ is the half-width of the cutting slot. The expression for a_i_ is given by $a_{i}=h_{i}\cdot \tan\left(\psi /2\right)$, where *c_li_* is the penetration depth of the i-th grain and the expression is shown in Equation (5) [27].

(5) $$c_{li}=k_{0}\cdot h_{i}$$

The expression for the coefficient k_0_ is given in Equation (6).

(6) $$k_{0}=\tan\left(\psi /2\right)\cdot \sqrt{\frac{3\left(1-2v\right)}{5-4v}+\frac{2\sqrt{3}}{\pi \left(5-4v\right)}\cdot \frac{E_{s}}{\sigma_{y}}\cdot \cot\left(\frac{\psi }{2}\right)}$$

According to Equations (1)–(6), the SSD depth is expressed as shown in Equation (7) [27]

(7) $$SSD=k_{1}\cdot h_{i}^{4/3}-k_{0}\cdot h_{i}$$

Currently, precise methods or models to estimate wafer substrate surface topography, particularly concerning material removal between grains and silicon, remain inadequate. Tao et al. [47] employed ultra-precision grinding on thin wafers, utilizing a laser confocal microscope to measure wheel surface and analyze grain protrusion heights. The measured heights, with mean and variance values of 0.39 and 0.14, respectively, closely matched simulated values of 0.47 and 0.20. These findings validate the model, optimizing grinding parameters for smoother wafer surfaces and advancing understanding of material removal in rotary grinding operations.

### 2.2. Diamond Wire Sawing by MAM

**Figure 4:**
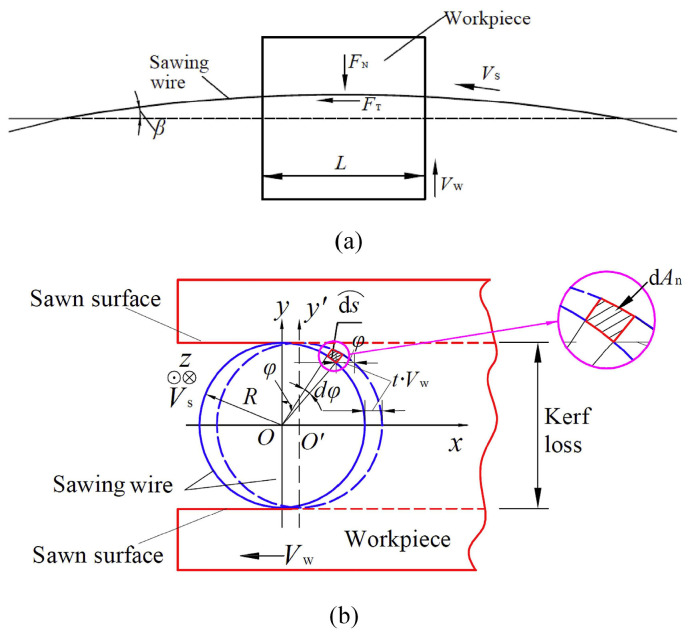
Theoretical model of fixed-abrasive wire sawing [50]: (**a**) a schematic of wire saw slicing and (**b**) a view of the sawing wire cross section.

Fixed abrasive wire sawing is vital for cutting and processing brittle materials and is widely used in industries including semiconductors and solar energy. Suzuki et al. [48] conducted wire sawing experiments on monocrystalline silicon and found that using finer diamond grits, increasing the number of cutting cycles, and enhancing wire speed significantly reduced surface roughness and SSD, thereby improving processing quality. These findings provide valuable insights for optimizing low-damage sawing processes. Chung et al. [49] employed numerical methods to simulate the wire sawing process. By mapping the cutting profile onto the workpiece model, they generated the surface topography of the cut material. The study investigated the effects of wire speed, feed rate, and the size and distribution of abrasive particles on the surface quality. The findings highlight the value of numerical methods in optimizing the cutting process. To further enhance understanding of material removal and surface generation during diamond sawing, examining theoretical models, as illustrated in Figure 4a, becomes crucial [50]. This theoretical framework helps in interpreting the observed experimental and simulation results and guides the optimization of the cutting process. The wire deflection angle during slicing is represented as β. *V_w_* denotes the workpiece’s feed speed, while *V_s_* represents the wire speed. Additionally, *R* corresponds to the nominal radius of the diamond wire, with the origin *O* situated at the center of the sawing wire’s cross-section. For coordinate axis establishment, the positive direction of the x-axis is opposite to the workpiece feed, the z-axis corresponds to the wire’s motion direction, and the y-axis, perpendicular to both the x and z axes, defined as angle ϕ within a range of 0–180°, specifies the location of diamond abrasives along the surface of the sawing wire, using the positive y-axis direction as the reference. If a single abrasive completes a cut from start to finish in time t, the average material thickness removed along the workpiece feed direction is t·*V_w_*. Equation (8) describes the sawing force, encompassing both normal and tangential components, exerted by an individual abrasive at different positions on the wire’s surface during the sawing process [50]:

(8) $$\left\{\begin{array}{c} F_{n}=\frac{2\sin\phi }{\pi \lambda n_{a}lR}F_{N}\\ F_{t}=\frac{\sin\phi }{2\lambda n_{a}lR}F_{T} \end{array}\right.$$

In the cutting process, *F_N_* and *F_T_* represent the respective definitions for the overall normal and tangential sawing forces, while λ denotes the proportion of active abrasive particles in the sawing process relative to the total abrasive quantity. *n_a_* represents the density of abrasive particles per unit area, and *l* denotes the length of the crystal being sawed. Expanding upon the sawing model presented in Figure 4 and described in Equation (8), it becomes evident that the force exerted by an individual abrasive particle located at the center of the kerf exceeds that of particles positioned near the sawn surface. Throughout the entire wire saw machining process, abrasives positioned at various locations along the circular direction of the saw surface will create varying degrees of indentation. Importantly, abrasives located at the center of the kerf remove a relatively larger volume of material, primarily contributing to material removal and kerf formation, thereby affecting cutting efficiency. Concurrently, abrasives located near the sawn surface primarily contribute to generating the surface texture of the sliced wafer and influencing the morphology of the sawn surface.

Utilizing the illustrated diagram in Figure 4b representing the wire sawing concept, the workpiece is advanced along the negative x-axis direction at a rate of V_w_·t during a specified time period, t. Taking the micro-arc $d_{ϒ∠}s$ at the position of angle ϕ in the *xy*-plane of the saw wire cross-section, the corresponding center angle of the micro-arc is dϕ, and the length of dsϒ∠ is determined as per the Equation (9) presented below [50]:

(9) dsϒ∠=R⋅dϕ

Approximately resembling a rectangle is the micro-area d*A*_n_, which is separated during a time span of *t* on the *xy* plane. The expression is shown in Equation (10) below [50]. This resemblance is particularly notable in the shadowed segment enclosed by red boundaries, as vividly presented in Figure 4b.

(10) $$\;dA_{n}=V_{W}\cdot t\;sin\phi \cdot^{ϒ∠}ds=V_{W}\cdot tR\cdot sin\phi d\phi$$

Given the length *L* of the workpiece that has undergone sawing, the volume d*V*_cut_ of material removed from the crystal within the time interval *t*, corresponding to the micro-area d*A*_n_, is expressed by the subsequent Equation (11) [50]:

(11) $$dV_{\mathrm{cut}}=L\cdot dA_{n}=L\cdot V_{W}\cdot tR\cdot \sin\phi d\phi$$

where *L* means the length of the sawn zone of the workpiece, assuming the deflection angle of the wire is constant during the whole sawing process.

**Figure 5:**
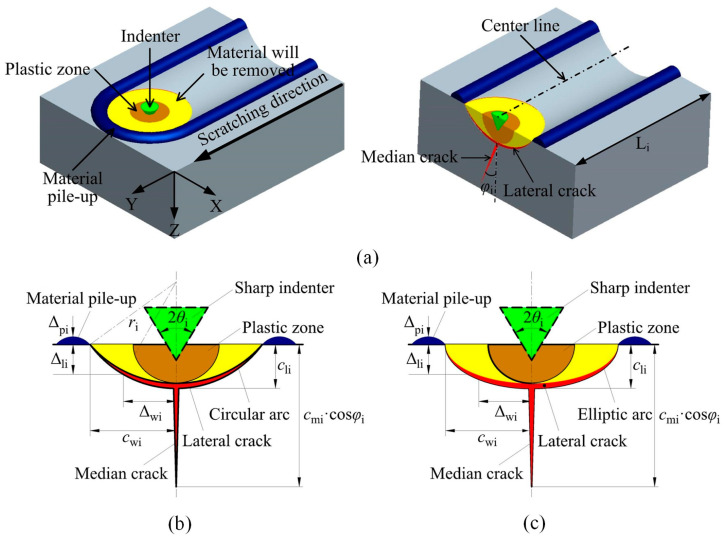
Fracture characteristics of brittle material under cone-shaped indentation [51]. (**a**) Notch made by a conical indenter on a brittle material; (**b**) assumed circular arc lateral crack system induced by cone-shaped indenter on brittle material; (**c**) elliptical arc lateral crack system induced by cone-shaped indenter on brittle material.

Damage to silicon wafers from diamond wire saws significantly impacts their mechanical, economic, and service properties, necessitating precise and swift evaluation. Yin et al. [51] developed a model to ascertain the depth of SSD based on the width of wafer fractures. During wire sawing, diamond abrasives generate scratches on the workpiece, with each abrasive cutting to depths of fractions of a micrometer. To simplify modeling and calculations, researchers approximated the abrasive cutting edge as conical. Figure 5a illustrates scratch grooves created by the conical indenter on brittle material, mimicking scratch formation on wafer surfaces. In ductile mode, material undergoes plastic deformation and is displaced from scratch grooves; in brittle mode, material is removed as debris, accumulating on both sides of scratch grooves and ahead of the indenter. The scratch resembles a series of indentations along its path. Figure 5b,c depict a typical intermediate–lateral crack system induced by indentation on brittle material. In brittle mode, material removal occurs through lateral crack formation, extending outward and eventually surfacing, resulting in brittle fractures manifested as fracture grooves and deep craters on the workpiece. Median cracks remain within the material, generating SSD. Fracture and SSD depths correlate with lengths of transverse and median cracks, respectively, with specific calculations detailed in Equations (12)–(16) presented below [52]:

(12) $$c_{li}=A\times F_{ni}^{1/2}$$

(13) $$SSD_{i}=c_{mi}\cos\varphi_{i}=(B\times F_{ni}^{2/3})\times \cos\varphi_{i}$$

(14) $$A=0.43{\left(\sin\theta_{i}\right)}^{1/2}{\left(\cot\theta_{i}\right)}^{1/3}\frac{E^{1/2}}{H}$$

(15) $$B={\left(\frac{\kappa k_{0}k_{1}^{2/3}}{k_{2}}\right)}^{2/3}{\left(\cot\theta_{i}\right)}^{4/9}\frac{E^{1/3}}{H^{1/3}K_{c}^{2/3}}$$

(16) $$\varphi_{i}=-1.5995\times \theta_{i}+133.13$$

where *c_li_* and *c_mi_* are the transverse crack length and midline crack length, respectively; SSD_i_ is the SSD depth; $\varphi_{i}$ is the midline crack deflection angle; $\theta_{i}$ is the half outer angle of the indenter; E, H, and K_c_ are the modulus of elasticity, indentation hardness, and fracture toughness of the material, respectively; *F_ni_* is the normal force; the subscript i stands for the ith parameter; κ is the modification coefficient; κ = 2.231; k_0_ is the dimensionless constant; and k_0_ ≈ 0.096 for the conical indenter [53,54].

Marshall et al. [55] obtained the relationship between transverse crack depth and width:

(17) $$\frac{c_{wi}}{c_{li}}=c\times F_{ni}^{1/8}$$

(18) $$C={\left(\cot\theta_{i}\right)}^{1/12}\frac{H^{1/2}}{E^{1/8}K_{c}^{1/2}{\left(1-\nu^{2}\right)}^{1/4}}$$

where *c_wi_* is the transverse crack width, which can be equal to the fracture width, and ν is the Poisson’s ratio of the material.

Comparing Equation (12) with Equation (18), it can be obtained that

(19) $$c_{li}=\alpha_{1}c_{wi}^{4/5}$$

(20) $$SSD_{i}=c_{mi}=\beta_{1}c_{wi}^{16/15}$$

where

(21) $$\alpha_{1}=\frac{A^{1/5}}{C^{4/5}}$$

(22) $$\beta_{1}=\frac{B\cos\varphi_{i}}{{\left(A\times C\right)}^{16/15}}$$

where parameters $\alpha_{1}$ and $\beta_{1}$ are influenced by the parameters of the indenter and the material properties.

Considering the influence of coolant performance, machine vibration, and other factors, two correction factors, $\alpha_{2}$ and $\beta_{2}$, are introduced in Equations (19) and (20), respectively:

(23) $$c_{li}=\alpha_{1}\alpha_{2}c_{wi}^{4/5}$$

(24) $$SSD_{i}=c_{mi}=\beta_{1}\beta_{2}c_{wi}^{16/15}$$

where the values of $\alpha_{2}$ and $\beta_{2}$ are fixed after the machine tool and coolant are determined; the values of $\alpha_{1}$, $\alpha_{2}$, $\beta_{1}$, and $\beta_{2}$ are obtained experimentally. Equations (23) and (24) show that the fracture depth and SSD depth can be determined by the fracture width and increase with the fracture width.

Then, many wafers were processed under different cutting parameters and the fracture parameters of these wafers were measured by confocal microscopy and the SSD depths were measured by cross-section scanning microscopy method. The calculated and measured SSD depths were analyzed in comparison. The results show that the model is able to accurately and quickly determine the SSD depth within 20 s with an average relative error of less than 12.0%.

### 2.3. Summary of MAM

The characteristics of the MAMs for simulating monocrystalline processing are summarized in Table 1. The investigation of the DWS of monocrystalline silicon is crucial in exploring its underlying mechanisms using MAMs. Through the examination of processes like grinding and wire cutting, we gain insights into how diamond particles influence material removal and surface morphology in silicon crystals during wire sawing. The application of mathematical models not only aids in optimizing process parameters for enhanced efficiency but also elucidates critical physical phenomena during the cutting process, such as calculating scratch depth and material removal rate. However, the modeling process involves idealizing actual conditions and simplifying complex phenomena, necessitating further refinement and validation.

## 3. Molecular Dynamics Model

MD is a computational simulation technique employed to analyze the physical movements of atoms and molecules comprising a system [56]. MD simulations meet the criteria for being atomic-scale simulations that depict the interactions of these atoms and molecules over time, offering insights into the dynamic evolution of the system [57,58,59]. The atomic foundation of MD simulations concurrently offers novel insights into the microscopic deformation mechanisms of materials, enabling the capture of changes in the position, velocity, stress, and energy information for each atom or particle [60,61].

### 3.1. Principles of MD

#### 3.1.1. Fundamental Principle

MD is crucial in examining nanoscale material removal [62,63,64]. The simulation begins by defining model parameters, molecular positions, velocities, and time steps. Potential functions are used to compute intermolecular interactions, while Newton’s equations are employed to update positions and velocities [65,66]. MD operates under classical mechanics, utilizing Coulomb’s law and Newton’s equations, and assumes that: (i) particles adhere to Newtonian mechanics and (ii) interactions follow the principle of superposition. Quantum effects and core electron interactions are not considered. Atomic positions are calculated using Newton’s equations, with forces derived from Equation (25) [67]:

(25) $$F_{i}{=m}_{i}\frac{d^{2}r_{i}}{dt^{2}}$$

In Equation (25), $F_{i}$ represents the force on the i-th atom in the system, $m_{i}$ is the mass of the i-th atom, and $r_{i}$ is the position vector of the i-th atom.

During the calculation process, a series of discrete velocity and position coordinates can be generated to describe the spatial position of each atom of the system over time. Once only the potential energy function U(r) is determined, the *F_i_* acting on atom i-th is calculated as follows (in Equation (26)) [67]:

(26) $$F_{i}=-\nabla_{r_{i}}U\left(r_{1},\cdots ,r_{N}\right)=-\left(\frac{\partial U}{\partial x_{i}},\frac{\partial U}{\partial y_{i}},\frac{\partial U}{\partial z_{i}}\right)$$

In Equation (26), $-\nabla_{r_{ij}}$ is the gradient operator.

After calculating the forces by Equation (26), the velocity and position of the individual atoms can be found by bringing in the equations. The current atomic position vectors can be illustrated by Equation (27), which shows the process [68]:

(27) $$\vec{r(t+\Delta t)}=\vec{r}\left(t\right)+\vec{v}\left(t\right)\Delta t+\frac{1}{2!}\vec{a}\left(t\right)\Delta t^{2}+\frac{1}{3!}\frac{d^{3}\vec{r}\left(t\right)}{dt^{3}}\Delta t^{3}+\cdots$$

Equation (27) is called the time step and is calculated on the order of femtoseconds (fs), i.e., 10–15 fs.

#### 3.1.2. Potential Function

The Tersoff potential function is a simple and fast three-body model for accurately describing covalent bonding in materials [69,70]. Ameli et al. [71] use the modified Tersoff function and the analytical bond order potential to model atom interactions in monocrystalline silicon during nanocutting. Choosing the right potential function is crucial for precise microscopic simulations, and the Tersoff function, which accounts for bond angles and covalent effects, is particularly effective for materials with diamond lattice structures such as carbon, silicon, and germanium. Its mathematical expression is given in Equations (28)–(31) [72,73]:

(28) $$E=\sum_{i}E_{i}=\frac{1}{2}\sum_{i\neq j}V_{ij}$$

(29) $$\begin{array}{l} V_{ij}=f_{c}\left(r_{ij}\right)\left[a_{ij}f_{R}\left(r_{ij}\right)+b_{ij}f_{A}\left(r_{ij}\right)\right]\\ f_{R}\left(r_{ij}\right)=A\exp\left(-\lambda_{ij}r_{ij}\right)\\ f_{A}\left(r_{ij}\right)=-B\exp\left(-\mu_{ij}r_{ij}\right) \end{array}$$

(30) $$f_{c}\left(r_{ij}\right)=\left\{\begin{array}{l} 1,r_{ij}<R_{ij}\\ \frac{1}{2}+\frac{1}{2}\cos\left[\frac{\pi \left(r_{ij}-R_{ij}\right)}{\left(S_{ij}-R_{ij}\right)}\right],R_{ij}<r_{ij}<S_{ij}\\ 0,r_{ij}>S_{ij} \end{array}\right.$$

(31) $$\begin{array}{l} b_{ij}=\chi_{ij}{\left(1+\beta_{i}^{n_{i}}\zeta_{ij}^{n_{i}}\right)}^{-1/2n_{i}}\\ \zeta_{ij}=\sum_{k\neq i,j}f_{C}\left(r_{ik}\right)\omega_{ik}g\left(\theta_{ijk}\right) \end{array}$$

In Equations (28) and (29), E and $V_{ij}$ correspond to the total energy and the energy associated with all atomic bonds, respectively; the labels I, j, and k are used to denote the atoms within the system; $r_{\mathrm{ij}}$ represents the bond length; while $b_{\mathrm{ij}}$ signifies the bond order term. Additionally, $f_{R}$ stands for the repulsive pair potential, and $f_{A}$ represents the attractive pairing potential. In Equations (30) and (31), the smoothed cutoff function, designated as $f_{C}$, serves the purpose of constraining the potential’s range. Moreover, $\zeta_{ij}$ is used to represent the count of bonds linked to atom “i” excluding those connected to atom j; the constant parameter is denoted as A, B, R, S, λ, μ, β, n, c, d, h.

The Morse potential, offering greater accuracy and efficiency than the Tersoff potential for Si-C interactions [74], is preferred in MD simulations of monocrystalline silicon DWS. It models three interaction types: (i) within the silicon, (ii) between the specimen and the tool, and (iii) within the tool. Its suitability for diatomic molecules and low computational cost make it ideal for specimen–tool interactions [75,76]. It also effectively describes Si-C interactions, as shown in Equation (32) [77]:

(32) $$E_{Si-C}\left(r_{ij}\right)=D_{M}\left[e^{-2a\left(r_{ij}-r_{M}\right)}-2e^{-a\left(r_{ij}-r_{M}\right)}\right]$$

In Equation (32), the cohesion energy, elastic modulus, and interatomic equilibrium distance at the state of equilibrium are represented by $D_{M}$, a, and $r_{M}$, respectively.

The main criteria for selecting an integration method for the equations of motion are energy conservation, accurate reproduction of thermodynamic properties, and computational efficiency. Common algorithms for solving Newton’s equations include the Verlet and velocity–Verlet algorithms [78]. Verlet’s algorithm, which relies on a third-order Taylor expansion of position r(t) and includes both a backward and a forward step, is fundamental. Its formulation is provided in Equation (33) [79]:

(33) $$r\left(t+\Delta t\right)=2r\left(t\right)-r_{i}\left(t-\Delta t\right)+\frac{d^{2}r\left(t\right)}{dt^{2}}\Delta t^{2}$$

Although Verlet’s algorithm was an effective early method, it is prone to errors with very small time intervals [68] and lacks a velocity term, complicating the calculation of atomic velocities. To address this, the velocity–Verlet algorithm [80] was developed. It computes both velocity and position accurately and directly, offers reasonable stability, and is time-reversible. The velocity–Verlet algorithm has largely supplanted the original Verlet method. Its formulation is given in Equation (34) [68]:

(34) $$\begin{array}{l} r_{i}\left(t+\delta t\right)=r_{i}\left(t\right)+v_{i}\left(t\right)\delta t+\frac{1}{2}a_{i}\left(t\right)\delta t^{2}\\ v_{i}\left(t+\frac{1}{2}\delta t\right)=v_{i}\left(t\right)+\frac{1}{2}a_{i}\left(t\right)\delta t\\ v_{i}\left(t+\delta t\right)=v_{i}\left(t+\frac{1}{2}\delta t\right)+\frac{1}{2}a_{i}\left(t+\delta t\right)\delta t \end{array}$$

For MD simulations where velocity accuracy is less critical, the Verlet algorithm is preferred due to its single force evaluation, in contrast to the two evaluations required by the velocity–Verlet algorithm [81]. The Beeman algorithm, related to Verlet, necessitates storing separate values for $\vec{r_{i}}\left(t\right)$, $\vec{v_{i}}\left(t\right)$, $\vec{a_{i}}\left(t-\delta t\right)$ providing improved storage capacity and longer integration intervals. Its formulation is presented in Equation (35) [82]:

(35) $$\left\{\begin{array}{l} \vec{r_{i}}\left(t+\delta t\right)=\vec{r_{i}}\left(t\right)+\vec{v_{i}}\left(t\right)\delta t+\frac{1}{6}\left[4\vec{a_{i}}\left(t\right)-\vec{a_{i}}\left(t-\delta t\right)\right]\delta t^{2}\\ \vec{v_{i}}\left(t+\delta t\right)=\vec{v_{i}}\left(t\right)+\frac{1}{6}\left[2\vec{a_{i}}\left(t+\delta t\right)-5\vec{a_{i}}\left(t\right)-\vec{a_{i}}\left(t-\delta t\right)\right]\delta t \end{array}\right.$$

Predictive correction algorithms are commonly used for integrating equations of motion. Cai et al. [83] employed MD simulations to examine nano-scale ductile cutting of monocrystalline silicon with a diamond tool, focusing on “dynamic hard particles” and tool wear. They used Gear’s algorithm with a 1 fs time step, which involves three sequential steps outlined in Equations (36)–(38) [84]:

(36) $${Χ}_{n+1}={Χ}_{n-1}+2v_{n}\Delta t$$

(37) $$V_{n+1}=V_{n}+\frac{1}{2}M^{-1}\left(F\left({Χ}_{n}\right)+F\left({Χ}_{n+1}\right)\right)\Delta t$$

(38) $${Χ}_{n+1}={Χ}_{n}+\frac{1}{2}\left[V_{n+1}+V_{n}\right]\Delta t$$

Equation (36) provides an initial estimate of the position, which is used to calculate the force. Equations (37) and (38) are then employed to compute the new velocity and position. Subsequently, the force is recalculated, and the process is iterated to refine the estimates of both position and velocity.

#### 3.1.3. Boundary Conditions

Boundary conditions are crucial for controlling computational complexity in molecular models. While exact conditions ideally eliminate boundary effects, they are nonlocal, involving all boundary atoms and their histories [85]. In MD simulations, periodic boundary conditions (PBC) are frequently employed to simulate infinite systems, allowing a small cell to effectively represent a larger material block and reduce surface effects [86,87,88]. For simulating the cutting of monocrystalline silicon, larger models are used to mitigate boundary effects, though this results in increased computational time [89]. Although PBC is suitable for homogeneous systems, it is inadequate for multiscale atomic continuum models with nonuniform regions. Zhou et al. [90] proposed a fitting equation to model changes in boundary forces for fluid states, as detailed in Equation (39) [90]:

(39) $$F_{b}=\left\{\begin{array}{l} p_{1}+p_{2}e^{{\left(r_{w}+0.25\right)}^{3.4}}\cos\left(p_{3}r_{w}\right),r_{w}\leq 1.04\sigma \\ \frac{-1}{q_{1}+q_{2}{\left(2.5-r_{w}\right)}^{2}+\frac{q_{3}}{{\left(2.5-r_{w}\right)}^{2}}},r_{w}>1.04\sigma \end{array}\right.$$

In Equation (39), $p_{1},\;p_{2},\;p_{3}$ is a function of density and temperature, $q_{1},\;q_{2},\;q_{3}$ is a function of the density, and $r_{w}$ is the distance from atom i to boundary $\Gamma_{f}$.

Grinding is crucial in precision and ultra-precision machining, essential for achieving high surface quality, accuracy, and integrity [91]. MD simulations provide valuable insights into atomic-scale machining and grinding processes [92]. In ultra-precision grinding, especially nano-grinding, material is removed at the atomic or few-layer level, making experimental observation difficult. Thus, MD simulations are effective for understanding nano-scale material removal mechanisms [93,94]. Guo et al. [95] used MD simulations on a monocrystalline silicon sample to investigate multiple grinding operations. Initial grinding was set at 20 Å depth, with subsequent steps incremented by 5 Å, leading to a predominant amorphous silicon damage layer and reduced hardness and elastic modulus. This indicates that shallower grinding depths reduce damage layer thickness without additional structural damage. Li et al. [96] examined the effect of grinding speed on SSD using MD simulations. Their results showed subsurface damage layer (SDL) thickness increased with grinding speed up to 100 m/s, then decreased, with a notable rise at 250 m/s. Optimal grinding speed is crucial in controlling SDL thickness and improving workpiece performance.

**Figure 6:**
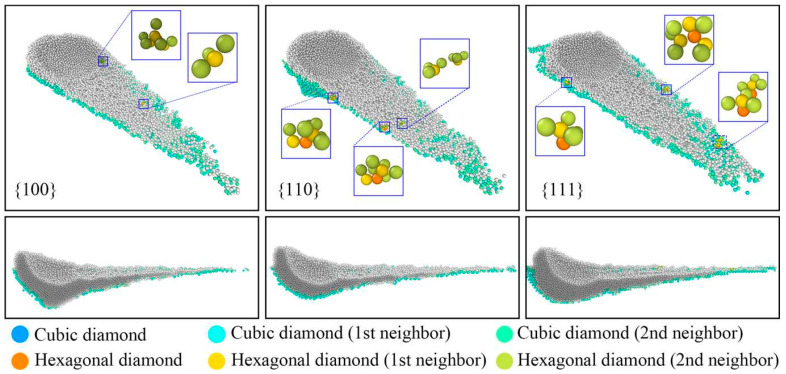
SDL and local magnification during processing in {100}, {110}, and {111} crystal orientations [98].

In the field of ultra-precision machining, the crystallographic orientation of a crystalline material’s surface exerts substantial influence on both surface formation and SSD. Zhao et al. [97] used MD simulations to study surface formation and subsurface damage in nano-grinding of monocrystalline silicon with different crystal orientations. While surface quality was similar for orientations ({100}, {110}, {111}), subsurface damage, processing forces, phase transformations, and residual stresses varied significantly. Higher processing forces and temperatures increased the thickness of the SDL, the number of phase-transformed atoms, and residual stresses. Workpieces with the {111} orientation had the most severe subsurface damage, while those with the {110} orientation had the least. Consequently, {110} orientation workpieces exhibited superior surface and subsurface quality. Further, MD simulations were utilized by Zhao et al. [98] to examine the grinding performance of silicon workpieces that are single-crystal and have varying crystal orientations. Figure 6 shows the schematics of the SDL and local magnification during processing for different crystal orientations. The {111} orientation contained the maximum number of defect structures with coordination numbers surpassing 7, and it displayed the most prominent phase transformation in the SDL when compared to the {100} and {110} orientations. In order to improve processing quality, this work offers atomic-level insights into choosing silicon wafers’ ideal crystal orientation.

### 3.2. Diamond Wire Sawing by MD

Since it is very difficult to directly observe and analyze the DWS of monocrystalline silicon at the atomic scale using experimental methods, MD simulation has become an essential tool for studying this complex process. Liu [68] developed a nanometer-scale simulation model of DWS of monocrystalline silicon using MD and conducted a thorough analysis of the machining mechanisms. The study indicated that the base of the diamond particles primarily removed silicon atoms from the workpiece through an extrusion process, while the sides of the particles created shallow cutting depths on the machined surface through scratching and plowing. The extrusion of diamond particles generated significant hydrostatic pressure and shear stress within the workpiece. When the hydrostatic pressure reached −5.6 GPa and the von Mises stress exceeded 10 GPa, silicon atoms transitioned directly from the crystalline phase to the amorphous phase. This research provides valuable theoretical guidance for optimizing the DWS process for monocrystalline silicon, enhancing machining accuracy, and achieving high-quality component surfaces.

**Figure 7:**
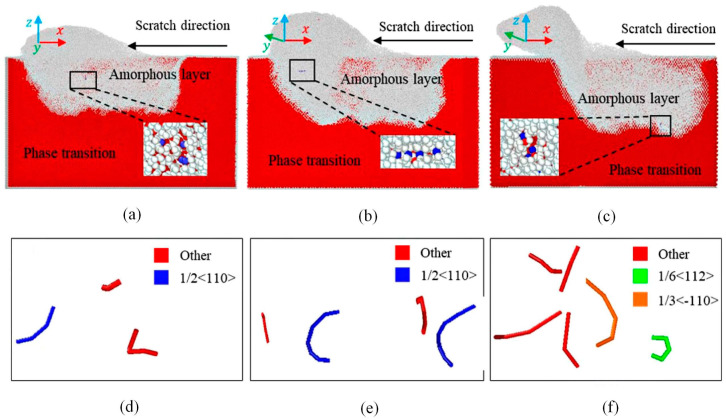
SDL and dislocation distributions in monocrystalline silicon [101]. SDL along the (**a**) [100], (**b**) [210], and (**c**) [110] zone axes; dislocation distributions along the (**d**) [100], (**e**) [210], and (**f**) [110] zone axes.

The DWS process for monocrystalline silicon is intricate and affected by numerous factors, including the mechanical properties of the material and the cutting conditions [99]. To optimize this process, understanding the scratching behavior of monocrystalline silicon is crucial. Using MD simulations, Niu et al. [100] examined the scratching behavior of {110} crystal oriented monocrystalline silicon at temperatures ranging from 1 K to 1000 K. They discovered that the high temperatures accelerated the buildup of silicon atoms on the tool’s front and flanks, improving the rate at which material was removed. In addition to temperature effects, Tao et al. [101] developed an MD simulation model of monocrystalline silicon scratching to reveal the diversity of dislocation and stress distribution under different crystal orientations. Figure 7a–c illustrate the SSD morphologies along different scratch directions, indicating that the deformation region induced by scratching consists of an amorphous layer and lattice defects. Figure 7d–f depict the dislocation distributions along different scratch directions. It is observed that the density of dislocation lines along the [100] zone axis is significantly lower than that along the [110] zone axis. Additionally, notable differences in the propagation direction and length of dislocations are observed under different scratch directions, suggesting that crystal orientation affects damage deformation. These studies provide a comprehensive theoretical foundation for understanding the mechanisms of material removal, stress distribution, and the influence of temperature and crystal orientation on the machining of anisotropic monocrystalline silicon, thereby supporting the optimization of DWS processes.

### 3.3. Summary of MD Model

The study of DWS of monocrystalline silicon using MD simulations is crucial for uncovering the atomic-scale mechanisms that drive the cutting process. By analyzing scratching behaviors and stress distributions, MD simulations provide valuable insights into material removal processes and the impact of various crystal orientations on cutting efficiency. These simulations help elucidate how silicon atoms deform and transition to an amorphous phase under conditions such as high hydrostatic pressure and shear stress, which aids in optimizing cutting parameters for better precision and surface quality. Additionally, MD simulations are computationally intensive and may not fully capture large-scale effects or variations in material properties encountered in practical applications. Therefore, while MD simulations are a powerful tool for understanding atomic-scale processes, their results should be complemented with experimental validation and ongoing refinement to improve their practical relevance and accuracy. The characteristics of the MD models for simulating monocrystalline processing are summarized in Table 2.

## 4. Finite Element Method Model

FEM is widely employed for modeling material deformation, providing approximate solutions to elliptic partial differential equations by dividing the domain and applying boundary conditions [102,103]. It consists of two main branches: one using discrete elements to determine displace-ments and forces in structural frames, and another employing continuous elements for heat transfer, fluid mechanics, and solid mechanics. Furthermore, by combining the physical modeling capabilities of FEM with the strengths of ML in addressing complex engineering problems, complex systems in different fields can potentially achieve a deeper understanding and optimization [104,105]. In machining simulations, FEM replicates various cutting conditions, optimizing tools and parameters by analyzing forces, stresses, damage, and other factors [106,107]. While computational limitations restrict the size of MD simulation models and affect the accurate representation of single-crystal silicon processing, FEM offers a balanced approach between efficiency and accuracy, making it effective for continuum medium mechanics [108,109].

### 4.1. Principles of FEM

FEM is a numerical analysis technique used to address mathematical models associated with practical engineering challenges. By solving these equations, FEM enables the prediction and analysis of the behavior and performance of materials, structures, and systems [110,111]. FEM transforms continuous problems into discrete problems, resulting in sparse sets of algebraic equations—either linear or nonlinear—that must be solved. The mathematical equation approach of FEM is similar to innovative technologies for performance enhancement, reflecting the trend of technology refinement in engineering and materials science [112,113,114]. This process, known as discretization, involves subdividing or approximating the solution domain into a combination of discrete elements [115,116]. The domain is divided into subunits or elements interconnected by nodes, some with fixed displacements and others subjected to specific loads [117,118]. In FEM analysis, the primary numerical outputs include nodal values of field variables and their derivatives, along with graphical representations such as curves, contours of field variables, and deformed shapes [117,119].

**Figure 8:**
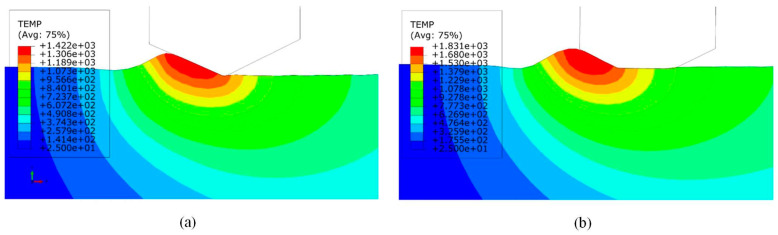
Software simulated cutting zone temperature map [120]. (**a**) Temperature distribution diagram before wear; (**b**) post-wear temperature profile.

High temperatures in monocrystalline silicon grinding worsen surface damage and tool wear. Understanding their impact is crucial in optimizing the process and extending tool life. An detailed investigation into the mechanisms causing surface degradation during monocrystalline silicon (100) single-point diamond grinding was carried out by Zhang et al. [120]. The temperature distribution within the grinding zone was evaluated using FEM simulations, and the results indicate that temperatures may rise above 1400 °C, as seen in Figure 8a. Furthermore, as seen in Figure 8b, the diamond abrasives flattened after grinding, losing their sharp cutting edges and raising the temperature in the cutting region. This model provided a thorough understanding of the grinding process by using FEM to investigate the temperature distribution and fluctuations of the diamond grinding wheel during the grinding of monocrystalline silicon.

### 4.2. Diamond Wire Sawing by FEM

**Figure 9:**
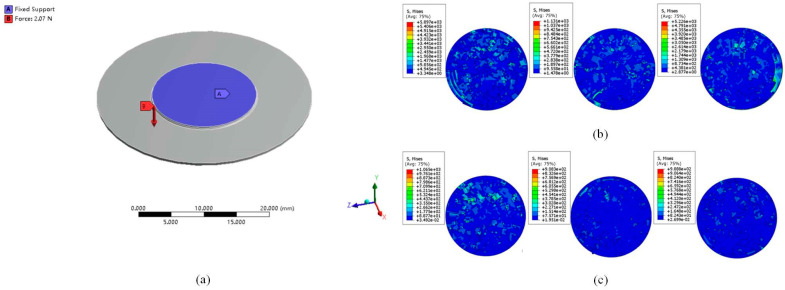
FEM simulation and analysis of wire-cut monocrystalline silicon. (**a**) Silicon wafer sawing equivalent simulation model [17]; (**b**) simulation results for h = 0.025, 0.035, 0.045 mm in CWS (*v_t_* = 2 m/s, *v_c_* = 1 mm/min, *n_w_* = 10 r/min) [123]; (**c**) simulation results for h = 0.025,0.035,0.045 mm in UAWS (*v_t_* = 2 m/s, *v_c_* = 1 mm/min, *n_w_* = 10 r/min) [123].

Silicon wafer thinning has become a prominent trend in the semiconductor industry, as it improves material utilization and reduces manufacturing costs [121]. However, a detailed theory to elucidate the machining mechanism for achieving the minimum thickness of monocrystalline silicon wafers using a diamond wire saw is currently lacking. Given the significant impact of this process on subsequent chip manufacturing technologies, Wang et al. [17] proposed a model based on Kirchhoff’s thin plate theory to analyze the minimum cutting thickness of silicon wafers using a diamond wire saw. Figure 9a presents the finite element analysis simulation model for silicon wafer cutting, demonstrating the model’s effectiveness. The simulation results reveal that increasing the cutting thickness of the silicon wafer leads to a reduction in equivalent maximum stress. The average deviation between the simulation results and the analytical model calculations is 9%. The study of abrasive wear effects on workpiece surface morphology is crucial in optimizing DWS processes, as it can enhance cutting parameters, accuracy, and surface finish. The effect of diamond wire wear on the surface morphology, roughness, and subsurface damage of single-crystal silicon wafers was evaluated experimentally by Kumar et al. [122]. The findings showed that the wafers showed higher ductile removal properties, decreased surface roughness, fewer but somewhat deeper subsurface cracks, and a lower average fracture strength as wire wear increased. By developing theoretical models, it is also vital to investigate how diamond rope saws cut monocrystalline silicon under various abrasive wear circumstances. Wang et al. [123] developed a FEM to simulate various abrasive wear conditions and analyzed the surface morphology of ultrasonic-assisted wire saws (UAWS) and conventional wire saws (CWS) under these conditions using finite element software, as shown in Figure 9b,c. At an abrasive wear value of h = 0.025 mm, the simulation showed an increase in brittle fractures and pits on the workpiece surface. At a wear value of 0.035 mm, the Mises stress decreased, indicating improved removal of ductile material and a reduction in the number of pits and grooves. This suggests that, beyond a certain level of abrasive wear, wire saws are more effective in removing ductile material and improving surface flatness. However, further increases in abrasive wear led to higher Mises stress values and a decline in surface quality. Comparative analysis of the Mises stress distribution and crater size for UAWS and CWS revealed that UAWS had lower stress values and smaller crater sizes compared to CWS. This indicates that high-frequency vibration assistance in UAWS reduces material removal time and minimizes chipping or fracturing at the workpiece edges. Consequently, ultrasonic vibration significantly enhances wire saw performance and workpiece surface quality.

**Figure 10:**
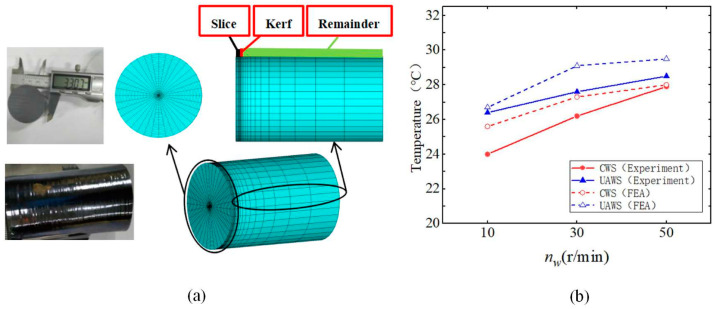
Finite element simulation of ultrasonic vibration-assisted wire sawing [126]. (**a**) Finite element model; (**b**) maximum cutting temperature at different speeds.

In fixed abrasive DWS technology, detecting the damage layer largely depends on experimental methods, which are both costly and time-consuming, and may also cause additional damage during the detection process. Zhang et al. [124] employed the FEM to investigate the multi-grain cutting process of monocrystalline silicon and evaluated the effects of wire speed, feed speed, and grain size on the damage layer through simulations. The results reveal that, at a wire speed of 10 m/s, the stress on the silicon surface changes significantly with variations in feed speed. When the feed speed is set at 0.194 mm/s, the damage layer depth is markedly influenced by the wire speed. Specifically, at a wire speed of 10 m/s and a feed speed of 0.194 mm/s, a decrease in grain distribution density results in an increased depth of the damage layer. In conclusion, to enhance surface quality, it is effective to increase both wire speed and grain distribution density while decreasing feed speed. Ultrasonic vibration technology has significantly improved both cutting efficiency and surface quality in silicon wafer processing. Several studies have demonstrated that ultrasonic vibrations hold potential for enhancing the quality of DWS monocrystalline silicon processing and other fields. [125,126]. Wang et al. [126] utilized ANSYS to establish a finite element model for ultrasonic vibration DWS of monocrystalline silicon, as illustrated in Figure 10a. The simulation focused on cutting temperatures. Figure 10b presents the maximum cutting temperatures obtained from both experimental and simulation results. It was found that, with an increase in workpiece rotational speed, the highest temperatures for CWS and UAWS were 27.9 °C and 28.5 °C, respectively. Additionally, the influence of cutting speed and wire speed on cutting temperature was examined. The average deviation between simulated and experimental temperatures was 8.6%, with a maximum deviation of 14%, thereby validating the accuracy of the model.

### 4.3. Summary of FEM Model

The use of FEM simulations in studying DWS of monocrystalline silicon is instrumental in optimizing cutting processes. FEM allows for detailed analysis of temperature distributions, stress variations, and tool wear during sawing, revealing critical insights into the effects of cutting parameters on material removal and surface quality. For instance, FEM helps to understand how variations in cutting thickness and wire speed impact stress levels and damage depth. This method also facilitates the evaluation of advanced techniques, such as ultrasonic-assisted sawing, and their influence on cutting efficiency and surface finish. While FEM simulations provide valuable theoretical guidance, their results must be validated through experimental studies to ensure practical applicability. Overall, FEM is a powerful tool for improving DWS processes and enhancing the precision and quality of silicon wafer cutting. The characteristics of the FEM models simulating monocrystalline silicon processing are summarized in Table 3.

## 5. Discussion

### 5.1. Similarity

MAM, MD simulations, and FEM models collectively aim to optimize the DWS process for monocrystalline silicon. MAMs provide theoretical predictions and insights into process parameters, MD simulations offer detailed atomic-scale information on material behavior and damage mechanisms, while FEM models evaluate macroscopic stresses, temperature distributions, and wear effects. Integrating the analyses from these models facilitates a comprehensive understanding of the wire sawing process, thereby aiding in the refinement and optimization of DWS for monocrystalline silicon. The similarities among these processing models are summarized in Table 4.

MAM, MD simulations, and FEM modeling each provide valuable insights into the DWS process for monocrystalline silicon. Although these methods vary in focus, ranging from theoretical predictions and atomic-scale details to macroscopic simulations, they share the common goal of optimizing the cutting process, understanding damage mechanisms, and enhancing surface quality. Integrating insights from these diverse approaches facilitates a comprehensive understanding of monocrystalline silicon processing and contributes to improved efficiency and performance in semiconductor fabrication.

### 5.2. Individuality

MAM provides theoretical predictions on how cutting parameters affect DWS but does not address detailed atomic or macroscopic material interactions. In contrast, MD simulations model atomic interactions to offer microscopic insights into material behavior and damage mechanisms, although they are computationally intensive and focus on smaller scales. FEM modeling, on the other hand, evaluates the effects of parameters on workpieces and tools by breaking down the cutting process into discrete elements and analyzing macroscopic stresses, temperature distributions, and wear effects. However, FEM does not provide the atomic-level detail seen in MD simulations or the theoretical insights offered by MAM. Table 5 summarizes the differences of the simulated monocrystalline silicon processing for the three types of models.

The distinctions between MAM, MD simulations, and FEM modeling arise from their varying methodological focuses and computational strategies. MAM employs mathematical formulas to predict the effects of cutting parameters on DWS; however, its reliance on simplified theoretical frameworks restricts its capacity to capture detailed atomic or macroscopic interactions. Conversely, MD simulations offer a microscopic perspective by modeling atomic interactions, providing a detailed analysis of material behavior and damage mechanisms, though they are computationally demanding and limited to smaller scales. FEM modeling addresses macroscopic stresses, temperature distributions, and wear effects through element discretization but lacks both the atomic-level detail of MD simulations and the theoretical depth of MAM. These disparities underscore the necessity for an integrated approach to thoroughly understand and optimize the DWS process for monocrystalline silicon.

### 5.3. Complementarity

MAM, MD simulations, and the FEM provide distinct insights into the DWS process for monocrystalline silicon, highlighting their complementary roles. MAM elucidates fundamental relationships within the DWS process through mathematical formulas that predict the effects of various cutting parameters. However, the theoretical predictions are limited by the inability to capture detailed atomic or macroscopic interactions. In contrast, MD simulations deliver a microscopic perspective by modeling atomic interactions, which reveal material behavior and damage mechanisms. Although computationally intensive and focused on smaller scales, MD simulations offer critical data that enhance the theoretical insights derived from MAM. Conversely, FEM, while lacking the theoretical depth of MAM and the atomic-level detail of MD simulations, provides a comprehensive analysis of how cutting parameters influence the workpiece and tool on a macroscopic scale. The integration of these approaches enables a thorough understanding of the monocrystalline silicon machining process, facilitating more effective optimization and enhancement of the cutting process.

## 6. Outlook

The DWS process for monocrystalline silicon can be effectively simulated by utilizing MAM, MD, and FEM models. More refinement is required to handle idealized situations, even while MAM models can optimize process parameters and explain physical phenomena like material removal rates and scratch depth. Experimental validation is necessary due to the computational intensity and limitations of MD simulations in capturing large-scale effects, despite the fact that they offer precise insights into material removal and the impacts of crystal orientation at the atomic scale. FEM simulations help with process optimization by analyzing temperature distributions and abrasive wear during the sawing process to ensure accuracy and practical application. But the theoretical inferences made by FEM simulations also need to be verified in practical settings.

The DWS process for monocrystalline silicon is better understood by using the combined insights provided by the MD simulations, FEM models, and MAM. MAM provides theoretical predictions, FEM assesses macroscopic stresses and temperature impacts, and MD simulations reveal atomic-scale information on material behavior. Although these models differ in scale and scope, they are all aimed at improving the cutting process, and when combined, they provide a thorough understanding that improves DWS’s accuracy and efficiency for monocrystalline silicon.

The future trends in the development of models for monocrystalline silicon processing may involve a deeper integration of models, combining different scales of modeling (MAMs, MD models, and FEM models) to comprehensively analyze and optimize the monocrystalline silicon processing at various levels.

(1) MAMs are poised to continue playing a crucial role in optimizing machining parameters and predicting process effects. By employing theoretical analysis and mathematical formulations, MAM can forecast temperature and stress distributions, optimize cutting parameters, reduce material damage, and enhance surface quality. In the future, MAM is expected to facilitate advanced process monitoring, feedback control, and cross-scale research in DWS of monocrystalline silicon, leading to significant improvements in cutting efficiency, quality, and stability. This advancement is likely to markedly enhance the technology for sawing monocrystalline silicon.
(2) The continuous advancements in computational power will facilitate the development of more detailed and accurate FEM models. These enhanced models will have the capability to capture intricate geometries and material behaviors, leading to a more realistic representation of monocrystalline silicon processing. FEM can further progress by integrating microstructure evolution across various processing stages. This extension allows for the prediction of imperfections, the evolution of grains, and various alterations at the microstructural level, all of which significantly influence material properties.
(3) In the future, integrating MD, FEM, and MAM in monocrystalline silicon processing will significantly enhance both understanding and optimization of the manufacturing process. MD models reveal microscopic phenomena such as atomic interactions, which can refine FEM models for improved macroscopic simulations. This leads to better machining strategies, temperature control, crystal growth rates, and management of related factors. Meanwhile, MAM provides quantitative optimization and theoretical support, facilitating precise process control and efficient production strategies, thereby advancing the precision and efficiency of silicon processing technology.
(4) Artificial Intelligence (AI) will play an important role in the study of DWS monocrystalline silicon through deep learning and optimization algorithms. AI can combine MD, FEM, and MAM to automatically analyze and optimize complex data from the cutting process. By learning from large amounts of experimental data, AI can reveal patterns of material behavior at the micro to macro level and automatically adjust model parameters to improve prediction accuracy.

## Figures and Tables

**Table 1:** MAM to simulate the processing characteristics of monocrystalline silicon.

Authors, Year	Purpose	Findings	Remarks
Li et al. 2017 [27]	To analyze grinding-induced SSD and surface roughness.	This model rapidly assessed SSD non-destructively.	The model measures silicon depth in grinding.
Tao et al. 2022 [47]	A novel 3D model revealed material removal and surface generation.	Errors were 5.3% and 12.9% for rough grinding, 7.3% and 15.6% for finish grinding.	The model reveals mechanisms in wafer spin grinding.
Gao et al. 2023 [50]	Theoretical analysis of cut depth and removal mechanisms in wire sawing.	Cut depth and roughness increased nonlinearly with the feed-to-wire speed ratio.	This study optimizes precision cutting parameters for silicon wafers.
Yin et al. 2019 [51]	A theoretical model determined SSD in monocrystalline silicon.	The model determined SSD depth in 20 s with a <12.0% error.	This research will improve wafer quality.

**Table 2:** MD to simulate the processing characteristics of monocrystalline silicon.

Authors, Year	Purpose	Findings	Remarks
Guo et al. 2016 [95]	Examined the effect of multiple millings on SDL thickness in silicon.	Reduced hardness and elasticity of machined surfaces.	Controlling damage layer thickness improves grinding quality.
Li et al. 2021 [96]	Analyzed damage mechanisms in silicon at different grinding speeds.	Analyzed the impact of processing parameters on silicon.	Reveals grinding speed’s effect on silicon SSD.
Zhao et al. 2022 [97]	Investigated milling of silicon with different surface orientations.	Observed variations in SSD, forces, phase transitions, and stresses.	Reveals crystal structure and SSD mechanisms by orientation.
Liu 2022 [68]	Analyzed DWS mechanisms for monocrystalline silicon.	The study uncovered silicon removal and phase transformation in DWS.	Provides theoretical guidance for optimizing DWS of silicon.

**Table 3:** FEM model to simulate the processing characteristics of monocrystalline silicon.

Authors, Year	Purpose	Findings	Remarks
Zhang et al. 2018 [120]	Investigated surface damage mechanisms of silicon (100) in diamond grinding.	FEM analyzed grinding zone temperature.	Reveals a new diamond grinding damage mechanism.
Wang et al. 2023 [17]	Investigated minimum thickness in silicon wafer machining.	Average simulation error for minimum sawing thickness was 9%.	Improves material use by studying minimum sawing thickness.
Zhang et al. 2010 [124]	Evaluated the effects of line speed, feed rate, and grain size on the damage layer.	Lower grain density increased damage layer depth at 10 m/s and 0.194 mm/s.	FEM analysis improves surface quality in silicon cutting.
Wang et al. 2021 [126]	Studied ultrasonic vibration effects on sawing temperature.	Validated simulation accuracy with 8.6% average and 14% maximum deviation.	Ultrasonic assistance has minimal temperature effect.

**Table 4:** Similarities in the processing of monocrystalline silicon by three types of models.

Models	Principle	Calculation Method	Presentation of Simulation Results	Remarks
MAMs	Mathematical analysis solves complex problems and models situations.	Equations, formulas, functions.	Numerical data, charts, graphs, tables.	The model utilizes math tools to describe the wire sawing process.
MD models	Atomic-scale mechanical and thermal behavior.	Open source software packages such as LAMMPS and MPICH.	Dynamic/static figures, predicted data.	The MD model analyzes atomic motion over time.
FEM models	The material is discretized and analyzed for deformation and stress.	Commercial software such as ANSYS and ABAQUS.	Numerical data, charts.	It simulates cutting monocrystalline silicon using mathematical models and algorithms.

**Table 5:** Differences in the processing of monocrystalline silicon in three types of models.

Models	Principle	Verification Method	Advantages	Disadvantages
MAMs	Analyze macroscopic cutting behavior, including force and stress concentration.	Direct experimental verification	Quantitative data enhance the accuracy of the cutting analysis model.	Simplification can cause deviations; high calculation costs.
MD models	Dynamic material removal predicts machinability.	Validation difficulties	Microscopic observation and prediction of silicon removal and processing properties.	No direct validation; model construction is challenging.
FEM models	Study chip formation, predict cutting forces, and analyze residual stresses.	Direct experimental verification	High prediction accuracy and broad usage.	Limited research on material removal mechanisms.

## Data Availability

Not applicable.
